# Epidemiological Characteristics and Clinical Features of Patients Infected With the COVID-19 Virus in Nanchang, Jiangxi, China

**DOI:** 10.3389/fmed.2020.571069

**Published:** 2020-11-04

**Authors:** Jian-Ming Hong, Long-Hua Hu, Qiao-Shi Zhong, Long-Chuan Zhu, Ya-Ping Hang, Xue-Yao Fang, Hua-Bao Sun, Zhi-Hua Huang, Jianping Xu, Yan-Hui Chen

**Affiliations:** ^1^Clinical Laboratory, The Ninth Hospital of Nanchang, Nanchang, China; ^2^Jiangxi Provincial Key Laboratory of Medicine, Clinical Laboratory, The Second Affiliated Hospital of Nanchang University, Nanchang, China; ^3^Infectious Diseases Department, The Ninth Hospital of Nanchang, Nanchang, China; ^4^Medical Imaging Department, The Ninth Hospital of Nanchang, Nanchang, China; ^5^Department of Biology, Michael G. DeGroote School of Medicine, Institute of Infectious Disease Research, McMaster University, Hamilton, ON, Canada

**Keywords:** COVID-19, cytokines, asymptomatic, epidemiogy, close contact, biochemical & physiological biomarkers

## Abstract

**Objectives:** The 2019 novel coronavirus disease (COVID-19) pandemic is the biggest public health crises in the 21st century. While most patients infected with the COVID-19 virus have no to moderate symptoms, there is currently limited clinical information about these patients.

**Methods:** In this study, we retrospectively investigated 41 patients infected with the COVID-19 virus in Nanchang, Jiangxi province, China, from February 4 to March 2, 2020. Nanchang is about 260 km southeast of Wuhan, the initial epicenter of the COVID-19 pandemic. We retrieved information on patient demographics, physical examination results, epidemiology, clinical manifestations, underlying conditions, laboratory analyses, radiological images, and treatment outcomes.

**Results:** Most patients (70.7%) had a history of close contact with patients with confirmed COVID-19, and 16 patients (39.0%) showed a high degree of family clustering. All 41 patients had no to moderate symptoms. The median age was 39.9 years and common symptoms of illness were fever (69.2%), cough (65.4%), and fatigue (19.2%). The dominant patient group was middle-aged women, with hypertension (14.6%) and chronic liver disease (12.2%) as the most frequent underlying conditions. All patients recovered, with the mean time of viral nucleic acid clearance at 10.6 days. Chest CT scans presented ground-glass opacities in 53.7% of patients while 26.8% had normal CT images. Laboratory results showed that lymphocyte counts, lymphocyte percentages, ESR, CRP, IgG, Fib, and cytokines were correlated to patients' conditions. Approximately 60–90% of patients had abnormally high levels of cytokines IL-4, IL-6, IL-10, and/or TNF-α.

**Conclusions:** Our results showed variable clinical and laboratory presentations among this group of patients infected with the COVID-19 virus. Though all 41 patients recovered, our results suggest that cytokine levels and other biochemical indicators should be monitored for patients infected with the COVID-19 virus showing no to moderate symptoms to ensure quick access for critical medical attention, if needed.

## Introduction

The 2019 coronavirus disease (COVID-19) was first identified in December 2019 in Wuhan, China (1). It's caused by the severe acute respiratory syndrome coronavirus 2 (SARS-CoV-2), commonly known as the COVID-19 virus (2, 3). Since then, the virus has spread globally, resulting in an ongoing pandemic. As of June 4, 2020, over 6 million cases have been reported across 216 countries and territories, resulting in more than 382,000 deaths (4). Within China where the pandemic originated, there have been 84,614 confirmed cases of infection by the COVID-19 virus (data on June 4, 2020) (5). Among the reported cases in China, about 20% of the patients had severe or critical illness, with the remaining 80% showing moderate to no symptom (5). A similar pattern is found globally where over 80% of infected patients have shown moderate to no symptom (4). Among the total of 84,614 cases in China, 932 were reported from Jiangxi (6), a province immediately to the southeast of Hubei province where the pandemic originated. Among the 932 cases in Jiangxi, 931 have since recovered and one patient died (6). There has been no active COVID-19 indigenous case in Jiangxi province since February 27, 2020, except for two imported cases from abroad on March 21, 2020 and March 28, 2020, respectively.

Based on sequence analyses, the Coronaviridae Study Group (CSG) of the International Committee on Taxonomy of Viruses revealed that the virus causing COVID-19 was similar to that causing the severe acute respiratory syndrome (SARS) epidemic in 2003 (7, 8). However, unlike patients infected with SARS-CoV, those infected with the COVID-19 virus typically have a longer incubation period, and with the majority of infected patients showing apparently no to moderate symptoms (9, 10). A large number of studies have shown that these asymptomatic to moderate cases have contributed significantly to the transmission and spread of the virus in communities throughout the world (11, 12). Indeed, the large proportion of asymptomatic carriers with no to moderate symptoms represents among the most difficult challenges to the prevention and control of the pandemic. Thus, understanding the epidemiological and clinical features of these patients could help future prevention and control of COVID-19.

Several studies have investigated the epidemiology and clinical features of COVID-19 patients in China. Those studies have come from either Wuhan, Hubei province, the original epicenter of the outbreak or large metropolitan areas such as Beijing and Shenzhen where travel between Wuhan and these cities were very frequent in the initial phase of the outbreak (December 2019 to January 23, 2020) before the lockdown of Wuhan. In contrast, cases from other provinces, including Jiangxi, located immediate to the southeast of Hubei, have not been reported. Compared to Wuhan (a mega-transport hub in central China), Jiangxi province is relatively isolated, and not a typical travel destination for tourists, especially in winter months. Thus, even though it was close to Wuhan, the number of cases in Jiangxi province was relatively small, with a cumulative 932 cases in a population of 45.2 million, a prevalence of COVID-19 at ~0.002% and a case-fatality rate of 0.11% (1/932). At present, the epidemiological and clinical feature of COVID-19 in Jiangxi is not known. The objective of this study is to retrospectively analyze the epidemiological and clinical characteristics of patients infected with the COVID-19 virus in a designated hospital for treating COVID-19 patients in the capital of Jiangxi province, Nanchang.

## Materials and Methods

### Study Population

We retrospectively reviewed the medical records of inpatients between February 4, 2020 and March 2, 2020 at the Ninth Hospital of Nanchang, the designated hospital for treating patients infected with the COVID-19 virus. For all patients, the diagnostic criteria followed the guidelines for diagnosis and treatment plan of COVID-19 issued by the National Health Commission (trial version 7) (13). Specifically, the tests were based on real-time, reverse-transcription polymerase chain reaction using the primers and probes targeting the *ORF1ab* and *N* genes of the COVID-19 virus, as recommended by the Chinese Center for Disease Control and Prevention (14). There must be a positive laboratory test showing the respiratory specimens containing nucleic acid of the COVID-19 virus in order for the patient to be included in this study.

### Ethics Statement

This work, which involved retrospectively reviewing medical records, was approved by the Ethics Committee of the Second Affiliated Hospital of Nanchang University, and the Ninth Hospital of Nanchang in Jiangxi province, China (License Number: 202004, Date: 13 March 2020). This was a retrospective study without intervention. However, when additional clinical specimens were needed for analyses, informed consent was obtained from all involved subjects.

### Data Collection

From the electronic medical record for each eligible patient, we extracted information on demographic characteristics (age, sex, and others), physical examination (highest temperature), epidemiology (Wuhan exposure history, history of contact with confirmed cases, and family and social gathering), and underlying disease conditions (high blood pressure, diabetes mellitus, chronic liver disease, and others). Clinical symptoms such as fever, cough, dyspnea, headache, fatigue, muscle ache, diarrhea, and others were obtained. Laboratory analytical results included white blood cell count (WBC), Lymphocyte percentage (Lym%), C-reactive protein (CRP), erythrocyte sedimentation rate (ESR), procalcitonin (PCT), heart function, liver and kidney function, coagulation test, cytokines test, immunoglobulin, cycle threshold (Ct) value in RT-qPCR, etc. Information on chest computerized tomography (CT), drug treatment, and prognosis of disease progression were also retrieved and evaluated. The virus clearance criteria were defined as two continuous negatives of nucleic acid tests according the guidelines for diagnosis and treatment plan of COVID-19 issued by the National Health Commission (trial version 7) (13). All of the patients' laboratory and radiological information were performed at admission, thus, some patients may have missing data.

### Case Classifications

All the retrieved cases for patients infected with the COVID-19 virus were classified into the following clinical categories: (i) Asymptomatic: no apparent symptom; (ii) Mild: mild fever without pneumonia manifestation through image results; (iii) Moderate: fever and other respiratory symptoms with pneumonia manifestation through image results; (iv) Severe: fever, respiratory distress with pneumonia manifestation through image results, hypoxia or abnormal results of blood gas analysis; (v) Critical: respiratory failure requiring mechanical ventilation, shock, or other organ failure requiring intensive care unit monitoring and treatment; and (vi) death.

### Statistical Analysis

For the statistical analysis, the associations between qualitative/categorical variables were analyzed using the Chi-square test or Fisher's exact test. Continuous variables were expressed as mean ± SD or median (interquartile range; IQR) and analyzed using Student's *t*-test, Mann–Whitney *U*-test or the Kruskal-Wallis *H*-test as appropriate. All tests were two-tailed, with *P* < 0.05 considered as statistically significant. All analyses were performed using SPSS version 20.0 for Windows (SPSS Inc., Chicago, IL, USA).

## Results

### Demographic Characteristics

Between February 4, 2020 and March 2, 2020, a total of 41 hospitalized patients were identified as infected by the COVID-19 virus at the Ninth Hospital of Nanchang. Of the 41 patients, 15 (36.6%), 7 (17.1%), and 19 (46.3%) were categorized into asymptomatic, mild, and moderate groups, respectively (Table 1). There was no severe or critical COVID-19 case in this hospital. All 41 individuals were identified as infected by the COVID-19 virus during their medical or home isolation. The median age of all patients was 39.9 years (range 15–83 years), with a female/male ratio of 1.56. Among the 41 patients, 5 (12.2%) were younger than 20 years old; 12 (29.3%) were between the ages of 21 and 40 years old; 19 (46.3%) were in patients aged from 41 to 60 years old; and 5 (12.2%) were aged 61 years and older (Table 1).

**Table 1:** Demographics, underlying diseases, and signs and symptoms on admission of 41 patients with COVID-19: 15 asymptomatic, 7 mild, and 19 moderate cases.

**Characteristics demographics**	**All cases** **(*n* = 41)**	**Asymptomatic cases** **(*n* = 15)**	**Mild cases** **(*n* = 7)**	**Moderate cases** **(*n* = 19)**
Sex ratio (F/M)	1.56	1.5	0.4	2.8
Age in years, mean (range)	39.9 (15–83)	42.7 (18–83)	30.6 (15–48)	41.2 (20–66)
**Age groups (years)**				
≤ 20	5 (12.2%)	2 (13.3%)	2 (28.6%)	1 (5.3%)
21–40	12 (29.3%)	4 (26.7%)	3 (42.8%)	5 (26.3%)
41–60	19 (46.3%)	6 (40.0%)	2 (28.6%)	11 (57.9%)
>60	5 (12.2%)	3 (20.0%)	0	2 (10.5%)
Underlying diseases	13 (31.7%)			
Hypertension	6 (14.6%)	2 (13.3%)	1 (14.3%)	3 (15.8%)
Diabetes mellitus	2 (4.9%)	1 (6.7%)	0	1 (5.3%)
Chronic liver disease	5 (12.2%)	2 (13.3%)	0	3 (15.8%)
**Signs and symptoms on admission**				
Fever	69.2% (18/26)	0	5 (71.4%)	13 (68.4%)
**Highest temperature**, **°C**				
37.3–38.0	44.4% (8/18)	0	80.0% (4/5)	30.8% (4/13)
38.1–39.0	44.4% (8/18)	0	20.0% (1/5)	53.8% (7/13)
>39.0	11.1% (2/18)	0	0	15.4% (2/13)
Cough	65.4% (17/26)	0	4 (57.1%)	13 (68.4%)
Fatigue	19.2% (5/26)	0	1 (14.3%)	4 (21.1%)
Dyspnea	7.7% (2/26)	0	0	2 (10.5%)
Muscle ache	7.7% (2/26)	0	0	2 (10.5%)
Headache	3.8% (1/26)	0	0	1 (5.3%)
Diarrhea	3.8% (1/26)	0	0	1 (5.3%)

The most common patient group was middle-aged women, with hypertension (14.6%) and chronic liver disease (12.2%) as the predominant underlying condition. Fever (69.2%), cough (65.4%), and fatigue (19.2%) are the common symptoms.

Data are presented as the median (range), or proportion (%), as appropriate.

Among the 41 patients infected with the COVID-19 virus, 9 (22.0%) had a history of recent travel to or living in Wuhan, and 29 patients (70.7%) had close contact with individuals with confirmed COVID-19 viral infection. Known family/social gathering accounted for 39.0% (16/41) of all cases. Of these 16 family/social clustering cases, 7 were due to family members returning from Wuhan, 6 had dinners together with people returning from Wuhan, and 3 unknowingly socialized with others who were later identified as having COVID-19. However, 3 of the 41 cases (7.3%) had no clear epidemiological history (Table 2). At least 13 of the 41 cases (31.7%) had underlying disease conditions, including 6 cases of hypertension, 2 cases of diabetes mellitus, and 5 cases of chronic liver disease. The other 28 (68.3%) patients had no known underlying condition (Table 1).

**Table 2:** Epidemiological history, chest CTs, and outcomes of 41 patients with COVID-19.

**Characteristics demographics**	**All cases** **(*n* = 41)**	**Asymptomatic cases** **(*n* = 15)**	**Mild cases** **(*n* = 7)**	**Moderate cases** **(*n* = 19)**
**Epidemiological history**				
Wuhan exposure history	9 (22.0%)	2 (13.3%)	2 (28.6%)	5 (26.3%)
Contact with confirmed cases history	29 (70.7%)	13 (86.7%)	3 (42.8%)	13 (68.4%)
No clear epidemiological history	3 (7.3%)	0	2 (28.6%)	1 (5.3%)
Family/social gathering	16 (39.0%)	6 (40.0%)	2 (28.6%)	8 (42.1%)
**Duration**				
Incubation period	9.1 ± 5.1	-	8.4 ± 2.7	8.7 ± 5.6
Duration from onset of symptoms to admission	4.2 ± 3.0	0	5.9 ± 4.3	3.6 ± 2.3
Duration of viral nucleic acid clearance	10.6 ± 4.2	9.7 ± 3.5	10.7 ± 4.6	11.2 ± 4.7
Duration of lesion absorption evidenced on CT images	13.7 ± 6.1	9.3 ± 4.9	-	16.4 ± 5.1
**Chest CT scan**				
Ground-glass opacity	22 (53.7%)	8 (53.3%)	0	14 (73.7%)
Focal lesion	5 (12.2%)	2 (13.3%)	0	3 (15.8%)
Bilateral lung patch shadow	3 (7.3%)	1 (6.7%)	0	2 (10.5%)
No abnormal lesion	11 (26.8%)	4 (26.7%)	7 (100.0%)	0
**Outcomes after treatment**				
Discharge	41 (100.0%)	15 (100.0%)	7 (100.0%)	19 (100.0%)
Secondary bacterial pneumonia	2 (4.9%)	0	1 (14.3%)	1 (5.3%)
Abnormal liver function	3 (7.3%)	1 (6.7%)	0	2 (10.5%)

Most patients (70.7%) had a history of close contact exposure with patients with confirmed COVID-19, and 16 patients (39.0%) were related to family/social clustering. Chest CT scans presented ground-glass opacity (53.7%), focal lesion (12.2%), bilateral lung patch shadow (7.3%), and normal CT image (26.8%). The mean time of viral nucleic acid clearance was 10.6 days.

*Data are presented as the percentage (%), or mean ± SD, as appropriate*.

### Clinical Characteristics

**Figure 1:**
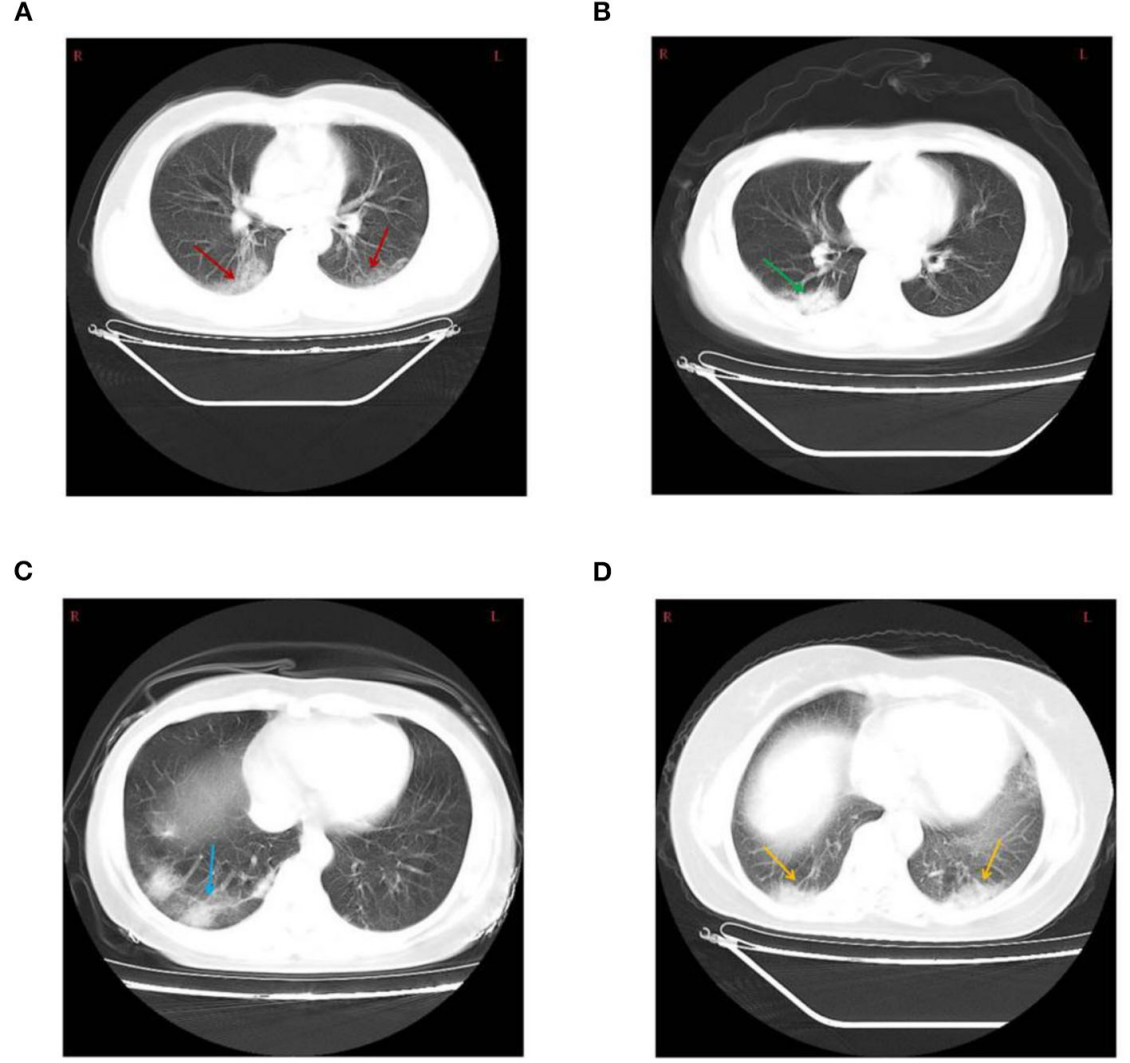
**(A)** Chest computed tomography showing bilateral diffuse ground-glass opacity (red arrow) in a 43-year-old male patient. **(B)** Patchy shadow of the right lung (green arrow) in a 44-year-old female patient. **(C)** Consolidation and Strip-shaped shadows of the right lung (blue arrow) in a 43-year-old female patient. **(D)** Bilateral lung patchy shadow (yellow arrow) in a 42-year-old female patient.

On admission to the Ninth Hospital of Nanchang, the most frequent symptom was fever (18/26, 69.2%) in the mild and moderate disease categories. For patients with fever, an equal proportion (at 44.4% each) suffered a low fever (37.3–38.0°C) and moderate fever (38.1 to 39.0°C). Two patients had high fever (>39°C). Cough (65.4%) and fatigue (19.2%) were also common symptoms in these patients. However, headache, dyspnea, muscle soreness, and diarrhea were relatively rare (Table 1). Seven patients were asymptomatic from exposure to admission, however, during the course of hospitalization, 2 asymptomatic ones developed mild fever and cough. Chest CT examination served as an important basis for the diagnosis of COVID-19 (Figure 1). Among the 41 patients, 22 showed ground-glass opacities, 5 cases showed focal lesions (patch or strip-shaped shadow), 3 cases showed bilateral lung patch shadow, and the remaining 11 showed no CT scan image abnormality. Interestingly, all seven mild cases showed normal CT scan images while 11 of the 15 asymptomatic cases showed abnormal CT scan images (Table 2).

### Treatment and Clinical Outcome

Among the 41 cases, the average incubation period was 9.1 days, with the longest being 20 days. The mean time from onset of symptoms to hospital admission was 4.2 days (Table 2). Antiviral therapy was given to 33 cases (80.5%) as the initial therapy which included lopinavir/ritonavir with ribavirin or arbidol. All 33 patients also took the traditional Chinese medicine Lotus Qingwen capsules as supplement to the antiviral therapy. The initial therapy followed the guidelines for diagnosis and treatment plan of COVID-19 issued by the National Health Commission (trial version 7) (13). In some cases, the patients also received antibiotic therapy, immunoglobin therapy, and/or bronchial nebulization of interferon-α2β. All 41 cases recovered and were discharged after two consecutive negative nucleic acid tests. They were then isolated at home for 14 additional days. None of the cases developed severe pneumonia, requiring systemic corticosteroids treatment, mechanical ventilation, or admission to ICU. In addition, there was no recurrence of clinical manifestations or positive nucleic acid tests in their follow-up visits at 2nd and 4th week after discharge. The duration of viral nucleic acid clearance, defined as the interval from the 1st day of positive nucleic acid tests to the two continuous negative tests, ranged from 4 to 20 days (mean: 10.6 days). The mean duration of lesion absorption as evidenced on CT scan was 13.7 ± 6.1 days. Secondary bacterial pneumonia was empirically diagnosed in two patients, one had a mild COVID-19 symptom and the other had a moderate symptom. However, neither patient had persistent pathology from bacterial infection and both patients recovered from the bacterial pneumonia after antibiotic treatment. Among the 41 cases, three (1 asymptomatic case and 2 moderate cases) had secondary abnormal liver function after antiviral therapy (Table 2).

### Laboratory Test Results

The laboratory results of the three groups of patients (asymptomatic, mild, and moderate cases) were summarized in Table 3, Supplementary Tables 1, 2. The results for blood routine markers, conventional inflammatory markers, cytokines, heart function, liver and kidney functions, immunity, and coagulation indicators are all presented in these tables as the median (IQR) or mean ± SD, as appropriate. The averages of the laboratory test results of 41 patients that showed above normal ranges included CRP, ESR, interleukin-4 (IL-4), interleukin-6 (IL-6), interleukin-10 (IL-10), interferon-γ (IFN-γ), tumor necrosis factor-α (TNF-α), and immunoglobulin G (IgG) (Supplementary Table 1). Statistical comparisons between asymptomatic cases and mild/moderate cases revealed that patients with relatively severe condition showed significantly higher levels of ESR, IL- 6, TNF-α, and fibrinogen (Fib) (*p* < 0.05) than the asymptomatic cases. However, no statistically significant difference was observed between the two groups of patients in their values of Lym%, CRP, IL-4, IL-10, IgG, or Ct-reading (Table 3).

**Table 3:** Statistical analyses of abnormal biochemical indicators in asymptomatic cases and mild/ moderate cases.

**Laboratory test results**	**Asymptomatic cases** **(*n* = 15)**	**Mild/moderate cases** **(*n* = 26)**	***P*-value**
**Blood routine test**			
Lym%, (20.0–40.0)%	20.6 ± 9.8	22.5 ± 9.6	0.59
<20%	6 (40.0%)	15 (57.7%)	0.34
**Conventional inflammatory markers**			
CRP, (0.0–10.0) mg/L	8.9 (3.9–13.8)	11.9 (5.0–15.9)	0.50
>10 mg/L	6 (40.0%)	15 (57.7%)	0.34
ESR, (0.0–20.0) mm/H	18.0 (13.5–23.5)	26.5 (20.0–56.2)	0.03
>20 mm/H	4 (26.7%)	15 (57.7%)	0.10
**Cytokines**			
IL-4, (0.0–2.80) pg/mL	5.9 ± 3.4	8.3 ± 4.5	0.09
>2.80 pg/mL	11 (73.3%)	24 (92.3%)	0.17
IL-6, (0.0–5.30) pg/mL	8.6 ± 7.3	18.6 ± 18.1	0.04
>5.30 pg/mL	9 (60.0%)	22 (84.6%)	0.13
IL-10, (0.0–4.91) pg/mL	8.9 ± 2.4	9.8 ± 7.5	0.07
>4.91 pg/mL	6 (40.0%)	19 (73.1%)	0.05
TNF-α, (0.0–2.31) pg/mL	3.2 ± 3.9	6.8 ± 6.5	0.02
>2.31 pg/mL	6 (40.0%)	19 (73.1%)	0.05
**Immune protein and complement**			
IgG, (7.0–16.0) g/L	15.5 ± 8.1	17.0 ± 10.1	0.36
>16.0 g/L	3 (20.0%)	8 (30.8%)	0.72
**Coagulation test**			
Fib, (2.0–4.0) g/L	2.7 ± 0.6	3.7 ± 1.1	0.03
>4.0 g/L	1 (6.7%)	11 (42.3%)	0.03
**RT-PCR**			
Ct-values	28.6 ± 5.1	28.5 ± 4.4	0.98

The patients with relatively severe condition showed significantly high levels of ESR, IL-6, TNF-α, and (Fib) (p < 0.05).

*Data are presented as the percentages (%), or mean ± SD, as appropriate. Lym%, Lymphocyte percentage; CRP, C-reactive protein; ESR, erythrocyte sedimentation rate; IL-4, interleukin-4; IL-6, interleukin-6; IL-10, interleukin-10; TNF-α, tumor necrosis factor-α; IgG, immunoglobulin G; Fib, fibrinogen; Ct-values, cycle threshold values*.

On admission, the majority of patients (37 of 41, 90.2%) had normal values of WBC counts. More than 50% of patients had lymphocyte counts (<0.8 × 10^9^ cells/L) and percentages (<20%) below the normal range in all three groups of patients (Supplementary Table 2). Between 60 and 90% of the patients had abnormally high cytokine levels in IL-4, IL-6, IL-10, and TNF-α (Supplementary Table 2). There were 21 (21 of 41, 51.2%) and 19 (19 of 41, 46.3%) patients with CRP and ESR above the normal range, respectively. 25–30% of the patients had abnormally high IgG (>16.0 g/L) and Fib (>4.0 g/L) levels (Supplementary Table 2). However, abnormal levels of PCT, lactate dehydrogenase (LDH), creatine kinase (CK), creatine kinase-MB (CKMB), myohemoglobin (MYO), cardiac troponin T (cTnT), aspartate aminotransferase (AST), alanine aminotransferase (ALT), total protein (TP), total bilirubin (TBIL), blood urea nitrogen (BUN), serum creatinine (Cr), immunoglobulin A (IgA), immunoglobulin M (IgM), complement 3 (C3), complement 4 (C4), prothrombin time (PT), Activation of partial prothrombin time (APTT), thrombin time (TT), and D-dimer (DD) were uncommon among the 41 patients (Supplementary Tables 1, 2).

## Discussion

In this study, we analyzed the demographic characteristics, physical examination results, underlying disease conditions, laboratory test results, and clinical characteristics of 41 patients infected with the COVID-19 virus who were hospitalized at the Ninth Hospital of Nanchang in Jiangxi Province, China. The 41 patients belonged to asymptomatic to mild and moderate cases, with no severe or critically ill cases. All 41 patients completely recovered. Our analyses identified that most patients had a history of close contact exposure with patients with confirmed COVID-19, including a high degree of family/social clustering. The most common patient group was middle-aged women, with hypertension and chronic liver disease as the predominant underlying disease conditions. The most common symptoms were fever and cough. Chest CT scans showed ground-glass opacities as the main type, followed by normal images, focal lesion and bilateral lung patch shadow. The laboratory results showed that in most COVID-19 cases, lymphocyte counts, lymphocyte percentages, ESR, CRP, IgG, Fib, and cytokines all deviated from the normal ranges. Below, we discuss the clinical significance of our results and their potential implications for the management of COVID-19.

In our study, 9 patients had a history of toured or lived in Wuhan in the early stage of the pandemic. These 9 patients were the primary cases in Nanchang, and most of these patients had mild to moderate symptoms. Twenty-nine of the 41 patients had close contact (e.g., living, dining, or socializing together) with confirmed primary COVID-19 patients in Nanchang. These 29 patients were the secondary cases and the inferred basic reproductive number (Ro) based on our observed cases is 3.22, consistent with published reports on COVID-19 (1, 9). Interestingly, the clinical manifestations of the secondary patients were mostly asymptomatic to mild, consistent with results from earlier studies (15, 16), which might have been due to their low-dose exposure to the virus and/or reduced virulence of the virus. For example, Chen et al. reported that the local cases in Chongqing were generally asymptomatic (16). Among our 41 cases, we were unable to identify the epidemiological history for 3 cases. The subsequent no new case in Jiangxi (including Nanchang) since February 27th (except the two imported cases) indicated that limiting the flow of people, prohibiting large gatherings, as well as encouraging people to stay at home and self-isolation were effective public health policies to reduce the spread of COVID-19 in Nanchang (17).

Our investigation identified more women (18) than men (16) infected with the COVID-19 virus in this hospital. This is different from several previous studies that showed more males than females with COVID-19 (1, 19, 20). The dominant patient group in our sample was in middle-aged people (30–50 years old), similar to the cohorts reported in other studies (16, 21–23). Interestingly, cases of the younger ages were more likely to be asymptomatic, or with mild symptoms, and to have a normal CT image. However, a few older individuals were also asymptomatic, including an 83-year-old in our patient sample. This result suggested that expanding the scope of nucleic acid testing to young people might be crucial to identify potential carriers to prevent the spread the COVID-19 and to reduce the risk of second wave of this pandemic (11, 16). Among the 41 patients, 13 (31.7%) had underlying disease conditions, including 6 with hypertension, 5 with chronic liver disease, and 2 with diabetes mellitus. Compared with several published studies, the proportion of our cases with underlying diseases was slightly higher (15, 21). However, the relatively small sample size of our study could have contributed to the slight difference.

The most common symptoms of patients with COVID-19 were fever (69.2%), however, high fever was rare. Cough was also common (65.4%), with most of the cough being dry cough without sputum. The other symptoms included fatigue, dyspnea, muscle ache, headache, and diarrhea, each at <20%. Our results are consistent with those reported in previous studies (24, 25). The average incubation period was 9.1 days and the longest was 20 days, higher than the 3–7 days announced by the National Health Commission. The asymptomatic carriers typically had no visible clinical manifestations on admission, and they often did not receive as much attention as symptomatic ones (22). However, it took a similar length of time for the asymptomatic patient group to clear viral nucleic acid as it was for the mild and moderate symptomatic groups (*P* = 0.61). Similarly, no correlation was found between Ct-value and disease severity, patient age, etc. Consequently, within the community, the asymptomatic cases could be a significant source of infections for other people. In the case of governments rushing to re-open the economy, public health should pay special attention to this group of people through active contact tracing and comprehensive health monitoring (11).

The most common chest CT scan of COVID-19 patients were ground-glass opacity (53.7%) (Figure 1), which is also the most common image of viral pneumonia (18). Interestingly, normal CT images were found in 26.8% of patients, with no evidence of pneumonia. Furthermore, we found that focal lesions were more likely to occur alone in the right lung, and most cases had variable lesions (Figure 1). Previous reports have shown that these lesions may be related to the severity and stage of COVID-19 disease progression (26). Overall, our results showed that care should be taken to use a combined set of criteria to diagnose the severity of COVID-19, separating it from other viral infections.

The mean time from illness onset to visit hospital was 4.2 days, consistent with Tian et al.'s study (27). The mean times of viral nucleic acid clearance and lesion absorptions as evidenced based on CT scan images were 10.6 and 13.7 days respectively. While we found no correlation between the time to viral nucleic acid clearance and time to lesion absorption, previous studies have shown that the lengths of time for both were positively associated with the severity of COVID-19 disease (21). Patients carrying the COVID-19 virus longer and with longer lesion absorption time typically have more severe clinical symptoms. In our cases, while all patients improved and were discharged from the hospital, and no cases of reinfection were found during the 30-day follow-up, a small number of patients developed secondary bacterial pneumonia and abnormal liver function during hospitalization, which required extended hospital stays. Thus, care must be taken to look for co-infections or secondary infections in COVID-19 patients. Indeed, there has been a suggestion that antibiotics should be used in COVID-19 patients to prevent secondary infections (19).

An increasing number of biomarkers have been identified as related to the severity of COVID-19 disease. For example, one study indicated that CRP and LDH might be good predictors of COVID-19 severity (23). Several researchers have also proposed the use of neutrophil-to-lymphocyte ratio as an independent risk factor for predicting the severity of COVID-19 (28). In addition, a recent study showed that abnormal coagulation results, especially markedly elevated D-dimer and FDP were common in deaths with severe COVID-19 (29). Consequently, guidelines for managing COVID-19 patients are continuously updated. The most recent COVID-19 diagnosis and treatment program (7th edition) published by the National Health Commission of China suggested using indicators such as low lymphocyte counts, high levels of CRP, ESR, CK, AST, and LDH, and high levels of cytokines in severe patients (13). In our study, the mean of most laboratory examinations at the time of admission for most participants with no to moderate symptoms were normal. However, a large proportion of patients had lymphocyte counts, lymphocyte percentages, and ESR, CRP, IgG, and Fib levels beyond the normal range. Notably, the levels of several cytokines such as IL-6, IL-10, and TNF-α increased dramatically after COVID-19 viral infection, with the degree of increases positively correlated with the severity of the disease. However, with patients improving, the cytokine levels gradually returned to normal. According to Ricardo et al. (30), the COVID-19 cytokine storms were likely the results of the interplay between inflammation and coagulation. Guidelines for the diagnosis and treatment of SARS-CoV-2 infected pneumonia were first published on January 30th, 2020, and it recommended that cytokine monitoring be applied to improve the curative rate and reduce mortality (31). Indeed, several studies showed that dynamic monitoring of cytokine levels, and several inflammatory biochemical indicators were important to predict the changes of patients' conditions and to carry out effective interventions as early as possible (19).

This research presented the clinical characteristics of patients infected with the COVID-19 virus in Nanchang, Jiangxi Province. Though we identified several important features in our data, our study has certain limitations. Specifically, our patient population was only part of the infected population in Nanchang and we were only able to collect data from patients who were either directly admitted to the Ninth Hospital of Nanchang or transferred from other hospitals to the Ninth Hospital of Nanchang. In addition, even though we analyzed all the known COVID-19 patients visiting our hospital, our patient population might not be representative of the entire COVID-19 patient population in Nanchang where severe/critical cases may be present in other hospitals in Nanchang. In order to obtain more accurate results, it would be better to conduct large-scale multicenter studies to extend the collection of more patient data in the province. Furthermore, the study data were retrospectively analyzed and mainly collected from patients at admission, with about 2% data missing. Additional information such as antibody testing for all patients should provide valuable information about the potential susceptibility of people with prior exposure to the COVID-19 virus.

## Conclusions

The COVID-19 pandemic is the biggest public health crises in the world. Various international and national public health organizations have been working hard to prevent and control the spread of the virus and effectively manage patients with COVID-19 viral infections. Having epidemiological and clinical information from early cases in China could help us better prepare strategies in other parts of the world, including the potential second wave. In this study, we conducted a comprehensive epidemiological and clinical analysis of 41 patients infected the COVID-19 virus in a specialized hospital designated for treating COVID-19 in Nanchang, Jiangxi province in China. Our analyses revealed that all 41 cases were asymptomatic, mild or moderate, and all have recovered. Most patients had a history of close contact exposure with patients with confirmed COVID-19 and showed a high degree of family clustering. Among these 41 patients, the most common group was middle-aged women. The most common symptoms were fever and dry cough with the majority of patients showing ground-glass opacity in their chest CT scan images. Laboratory analyses showed that most patients had abnormal lymphocyte counts, lymphocyte percentages, and levels of ESR, CRP, IgG, and Fib. Furthermore, the levels of several cytokines were significantly higher in these COVID-19 patients than the normal ranges for healthy individuals, and with the degree of cytokine elevation positively correlated with the severity of the disease. Interestingly, we found little differences in most biochemical test results among the asymptomatic, mild, and moderate group of cases. Our results suggest the close monitoring of cytokine levels and other inflammatory and biochemical indicators should be conducted for all infected patients to help determine their medical needs. Further follow-up studies are needed to determine whether the infected patients are immune to future infections by the COVID-19 virus.

## Data Availability Statement

The raw data supporting the conclusions of this article will be made available by the authors, without undue reservation.

## Ethics Statement

The studies involving human participants were reviewed and approved by The study was approved by the ethics committee of the Second Affiliated Hospital of Nanchang University, the Ninth Hospital of Nanchang in Jiangxi province, China, and written informed consent was obtained from the patients or their next of kin (License Number: 202004, Date: 13 March 2020). Written informed consent to participate in this study was provided by the participants' legal guardian/next of kin.

## Author Contributions

J-MH and Y-HC participated in the study design and conceptualization. J-MH, L-CZ, H-BS, and Z-HH participated in the acquisition of data. L-HH, Q-SZ, Y-PH, and X-YF participated in analysis and interpretation of data. J-MH and L-HH participated in drafting of the manuscript and participated in the statistical analysis. J-MH, L-HH, Y-HC, and J-PX participated in critical revision of the manuscript for important intellectual content. Y-HC and J-PX participated in administrative, technical, material support, and participated in study supervision. All authors contributed to the article and approved the submitted version.

## Conflict of Interest

The authors declare that the research was conducted in the absence of any commercial or financial relationships that could be construed as a potential conflict of interest.
