# Supplementary material for: Epidemiological Characteristics and Clinical Features of Patients Infected With the COVID-19 Virus in Nanchang, Jiangxi, China
Source: Front Med (Lausanne). 2020 Nov 4;7:571069. doi: 10.3389/fmed.2020.571069 (PMC7673370; doi:10.3389/fmed.2020.571069)
Supplement: Supplementary file 1 [file Data_Sheet_1.docx]

Supplementary Material

**TABLE S1.** Blood routine, conventional inflammatory markers, cytokines, heart function, liver and kidney function, immunity, and coagulation indicators of patients with COVID-19 viral infections. The mean of most laboratory examinations at the time of admission for participants with mild disease were normal. Only the following eight biochemical markers showed average values above the normal range: CRP, ESR, IL-4, IL-6, IL-10, IFN-γ, TNF-α, and IgG.

| **Laboratory test results** | **All cases (n=41)** | **Asymptomatic cases (n=15)** | **Mild cases (n=7)** | **Moderate cases (n=19)** |
| --- | --- | --- | --- | --- |
| **Blood routine test** |  |  |  |  |
| WBC, (4.0-10.0)×10^9^/L | 6.2±2.7 | 7.2±3.3 | 6.8±1.8 | 5.1±1.9 |
| Neutrophil, (2.0-7.7)×10^9^/L | 4.3±2.2 | 5.1±2.8 | 4.6±1.3 | 3.5±1.6 |
| Lymphocyte, (0.8-4.0)×10^9^/L | 1.2±0.6 | 1.2±0.4 | 1.6±0.7 | 1.1±0.6 |
| Lym%, (20.0-40.0)% | 21.8±9.6 | 20.6±9.8 | 23.2±8.7 | 22.3±9.6 |
| **Conventional inflammatory markers** |  |  |  |  |
| CRP, (0.0-10.0) mg/L | 10.4 (4.3-15.7) | 8.9 (3.9-13.8) | 3.5 (2.7-7.3) | 14.1 (7.1-20.0) |
| ESR, (0.0-20.0) mm/H | 20.0 (18.0-47.0) | 18.0 (13.5-23.5) | 20.0 (12.0-26.5) | 28.0 (20.0-61.0) |
| PCT, (0.0-5.0) ng/mL | 0.06 (0.04-0.08) | 0.06 (0.04-0.09) | 0.05 (0.04-0.06) | 0.06 (0.05-0.10) |
| **Cytokines** |  |  |  |  |
| IL-2, (0.0-5.71) pg/mL | 2.4±2.6 | 1.6±1.9 | 2.3±1.9 | 3.1±3.1 |
| IL-4, (0.0-2.80) pg/mL | 7.5±4.3 | 5.9±3.4 | 7.8±5.6 | 8.5±3.9 |
| IL-6, (0.0-5.30) pg/mL | 14.9±15.6 | 8.6±7.3 | 15.8±15.9 | 18.6±18.3 |
| IL-10, (0.0-4.91) pg/mL | 9.3±8.2 | 8.9±2.4 | 11.0±11.6 | 8.9±4.9 |
| IFN-γ, (0.0-7.42) pg/mL | 11.4±15.6 | 11.0±15.2 | 14.7±18.6 | 10.4±14.6 |
| TNF-α, (0.0-2.31) pg/mL | 5.5±5.9 | 3.2±3.9 | 6.3±5.7 | 6.9±6.6 |
| **Cardiac function index** |  |  |  |  |
| LDH, (109.0-245.0) U/L | 192.5±67.2 | 162.8±23.9 | 178.7±25.3 | 220.9±86.6 |
| CK, (0.0-190.0) U/L | 83.8±49.1 | 78.2±25.8 | 98.0±36.7 | 82.9±63.8 |
| CKMB, (0.0-24.0) U/L | 11.9±5.3 | 9.6±2.7 | 11.8±5.8 | 13.9±5.7 |
| MYO, (0.0-58.0) ng/mL | 28.9±22.9 | 26.8±17.2 | 14.6±15.7 | 35.8±26.1 |
| cTnT, (0.0-0.3) ng/mL | 0.22±0.21 | 0.22±0.26 | 0.13±0.11 | 0.25±0.18 |
| **Liver function index** |  |  |  |  |
| AST, (0.0-40.0) U/L | 25.1±8.9 | 24.5±9.9 | 22.3±4.5 | 26.6±8.9 |
| ALT, (13.0-35.0) U/L | 31.3±28.3 | 33.2±33.3 | 30.2±19.5 | 30.2±26.5 |
| TP, (60.0-87.0) g/L | 74.1±4.1 | 74.1±3.9 | 75.9±5.0 | 73.3±3.7 |
| TBIL, (3.42-20.5) μmol/L | 10.5±4.7 | 9.6±4.1 | 12.9±4.8 | 10.3±4.8 |
| **Kidney function index** |  |  |  |  |
| Cr, (53.0-97.0) μmol/L | 67.1±15.6 | 67.4±15.8 | 66.8±11.0 | 67.0±16.8 |
| BUN, (2.9-6.42) mmol/L | 4.4±1.5 | 4.9±1.5 | 3.9±1.1 | 4.2±1.6 |
| **Immune protein and complement** |  |  |  |  |
| IgG, (7.0-16.0) g/L | 16.1±9.4 | 15.5±8.1 | 13.3±3.0 | 18.4±11.2 |
| IgA, (0.7-3.8) g/L | 2.9±1.1 | 2.9±1.5 | 2.9±1.1 | 3.0±0.8 |
| IgM, (0.4-2.6) g/L | 1.2±0.5 | 1.1±0.4 | 1.1±0.2 | 1.4±0.5 |
| C3, (0.8-1.6) g/L | 1.2±0.2 | 1.2±0.3 | 1.3±0.2 | 1.2±0.2 |
| C4, (0.1-0.4) g/L | 0.19±0.08 | 0.20±0.10 | 0.18±0.04 | 0.20±0.08 |
| **Coagulation test** |  |  |  |  |
| PT, (10.0-14.0) s | 11.6±0.6 | 11.3±0.5 | 11.7±0.6 | 11.8±0.5 |
| APTT, (28.0-43.0) s | 27.7±0.7 | 25.9±2.5 | 28.9±1.9 | 28.7±2.4 |
| TT, (14.0-21.0) s | 16.7±1.5 | 16.8±1.4 | 17.6±1.2 | 16.3±1.6 |
| DD, (0.0-0.6) mg/L | 0.4±0.4 | 0.3±0.1 | 0.2±0.1 | 0.4±0.5 |
| Fib, (2.0-4.0) g/L | 3.3±1.1 | 2.7±0.6 | 2.9±0.8 | 3.9±1.1 |

Data are presented as the median (IQR) or mean ± SD, as appropriate. WBC: blood cell count; PCT: procalcitonin; IL-2: interleukin-2; IFN-γ: interferon-γ; LDH: lactate dehydrogenase; CK: creatine kinase; CKMB: creatine kinase-MB; MYO: [myohemoglobin](file:///E:/Program%2520Files%2520(x86)/Youdao/Dict/8.8.0.0/resultui/html/index.html#/javascript:;); cTnT: cardiac troponin T; AST: aspartate aminotransferase; ALT: aspartate aminotransferase; TP: total protein; TBIL: [total](file:///E:/Program%2520Files%2520(x86)/Youdao/Dict/8.8.0.0/resultui/html/index.html#/javascript:;)  [bilirubin](file:///E:/Program%2520Files%2520(x86)/Youdao/Dict/8.8.0.0/resultui/html/index.html#/javascript:;); Cr: serum creatinine; BUN: blood urea nitrogen; IgA: immunoglobulin A; IgM: immunoglobulin M; C3:complement 3; C4: complement 4; PT: [prothrombin](file:///E:/Program%2520Files%2520(x86)/Youdao/Dict/8.8.0.0/resultui/html/index.html#/javascript:;) [time](file:///E:/Program%2520Files%2520(x86)/Youdao/Dict/8.8.0.0/resultui/html/index.html#/javascript:;); APTT: activation of partial prothrombin time; TT: thrombin time; DD: D-dimer;

**TABLE S2** The percentage of patients with abnormal biochemical indicators relative to the normal reference range. 90.2% patients had normal values of WBC counts; more than 50% of patients had lymphocyte counts and percentages below the normal range in the moderate case group; approximately 60% to 90% of patients had abnormal cytokine levels (IL-4, IL-6, IL-10, and TNF-α); 51.2% and 46.3% patients respectively had CRP and ESR above the normal ranges.

| **Laboratory test results** | **All cases (n=41)** | **Asymptomatic cases (n=15)** | **Mild cases (n=7)** | **Moderate cases (n=19)** |
| --- | --- | --- | --- | --- |
| **Blood routine test** |  |  |  |  |
| WBC, >10×10^9^/L | 4 (9.8%) | 2 (13.3%) | 1 (14.3%) | 1 (5.3%) |
| Lymphocyte, <0.8×10^9^/L | 15 (36.6%) | 4 (26.7%) | 1 (14.3%) | 10 (52.6%) |
| Lym%, <20% | 21 (51.2%) | 6 (40.0%) | 3 (42.8%) | 12 (63.2%) |
| **Conventional inflammatory markers** |  |  |  |  |
| CRP, >10 mg/L | 21 (51.2%) | 6 (40.0%) | 2 (28.6%) | 13 (68.4%) |
| ESR, >20 mm/H | 19 (46.3%) | 4 (26.7%) | 3 (42.8%) | 12 (63.2%) |
| **Cytokines** |  |  |  |  |
| IL-2, >5.71 pg/mL | 2 (4.9%) | 1 (6.7%) | 0 | 1 (5.3%) |
| IL-4, >2.80 pg/mL | 35 (85.4%) | 11 (73.3%) | 6 (85.7%) | 18 (94.7%) |
| IL-6, >5.30 pg/mL | 31 (75.6%) | 9 (60.0%) | 5 (71.4%) | 17 (89.5%) |
| IL-10, >4.91 pg/mL | 25 (61.0%) | 6 (40.0%) | 4 (57.1%) | 15 (78.9%) |
| IFN-γ, >7.42 pg/mL | 16 (39.0%) | 4 (26.7%) | 4 (57.1%) | 8 (42.1%) |
| TNF-α, >2.31 pg/mL | 25 (61.0%) | 6 (40.0%) | 4 (57.1%) | 15 (78.9%) |
| **Immune protein and complement** |  |  |  |  |
| IgG, >16.0 g/L | 11 (26.8%) | 3 (20.0%) | 2 (28.6%) | 6 (31.6%) |
| **Coagulation test** |  |  |  |  |
| Fib, >4.0 g/L | 12 (29.3%) | 1 (6.7%) | 1 (14.3%) | 10 (52.6%) |
